# Dynamics of ATP-dependent and ATP-independent steppings of myosin-V on actin: catch-bond characteristics

**DOI:** 10.1098/rsif.2020.0029

**Published:** 2020-04-08

**Authors:** Ping Xie

**Affiliations:** Institute of Physics, Chinese Academy of Sciences, Beijing 100190, People's Republic of China

**Keywords:** molecular motor, myosin-V, run length, unbinding rate, catch bond

## Abstract

An analytical theory is presented for the dynamics of myosin-V molecular motor, where both ATP-dependent and ATP-independent steppings are taken into account. Specifically, the dependences of velocity, run length and unbinding rate upon both forward and backward loads and ATP concentration are studied, explaining quantitatively the diverse available single-molecule data and providing predicted results. The results show that the unbinding rate increases with the increase of ATP concentration and levels off at both low and high ATP concentrations. More interestingly, at an ATP concentration that is not very low, the unbinding rate exhibits characteristics of a catch-slip bond under backward load, with the unbinding rate decreasing rapidly with the increase of the backward load in the range smaller than about 2.5 pN and then increasing slowly with the further increase of the backward load. By contrast, under forward load the unbinding rate exhibits a slip-bond characteristic.

## 1. Introduction

Myosin-V is a homodimeric molecular motor involved in various intracellular transport processes [1]. It can move processively on an actin filament. Under no or low external force or load, the processive stepping of the molecular motor toward the barbed or positive end of actin (the forward direction) is powered by the free energy released from the ATPase activity. The motor moves in a hand-over-hand manner, with a step size of about 36 nm and an unloaded velocity of about 500 nm s^−1^ at saturating ATP concentration [2–4].

Using high-resolution single-molecule optical trappings, it was determined that the stall force of myosin-V, which is defined as the external force under which the mean velocity is equal to zero, is around 3 pN [5,6]. The dependences of the velocity, dwell time between two mechanical steps and ratio of forward to backward steps (simply called stepping ratio) upon ATP concentration and external force smaller than the stall force were well determined [5,6]. The dependence of run length, which is defined as the distance travelled by an individual myosin-V on an actin filament before unbinding, upon ATP concentration and external force was also studied elaborately [7,8]. It was shown that in the range of ATP concentration larger than 10 µM, as the ATP concentration increases the unloaded run length decreases [7]. At saturating ATP concentration and under the external force in the range of –5 pN (forward force) to 1.5 pN (backward force), the run length is almost independent of the force although the velocity decreases evidently with the force [8]. More interestingly, Gebhardt *et al*. [9] found that besides the ATP-dependent processive stepping under the external force smaller than the stall force, myosin-V can also make processive backward stepping with a step size of about 36 nm under the superstall force, which is almost completely independent of the ATPase activity. Accordingly, the dependences of the velocity upon ATP concentration and the external force in the wide range from the forward force to the backward force larger than the stall force were also revealed [9].

Apart from the above-mentioned quantities such as the velocity, dwell time and run length, the unbinding rate of myosin-V from actin during its processive movement is another important quantity to characterize its dynamics. In addition, in order to study theoretically and computationally the collective transport of cargos by multiple myosin-V motors, besides the force–velocity relation of the single myosin-V motors the force–unbinding rate relation is also essential, the latter of which has not received attention.

Although the dependences of velocity of myosin-V upon ATP concentration and the external force in the range smaller than the stall force have been extensively studied theoretically and computationally [10–20], the theoretical study under the external force in the range larger than the stall force has not been paid much attention and the single-molecule data of Gebhardt *et al*. [9] have not been explained quantitatively. The single-molecule data of Clemen *et al*. [8], which showed that under the external force in the range of –5 pN (forward force) to 1.5 pN (backward force), while the velocity decreases evidently with the force the run length is almost independent of the force, have not been explained theoretically. Moreover, how the ATP concentration and external force affect the unbinding rate is unclear. The purpose of this work is to study theoretically the dynamics of myosin-V under the external force in a wide range from the forward force to the backward force larger than the stall force on the basis of our proposed model, addressing the above-mentioned unclear issues, which has strong implications for the stepping mechanism of myosin-V.

## 2. The model

### 2.1. The chemomechanical coupling pathway

**Figure 1.**
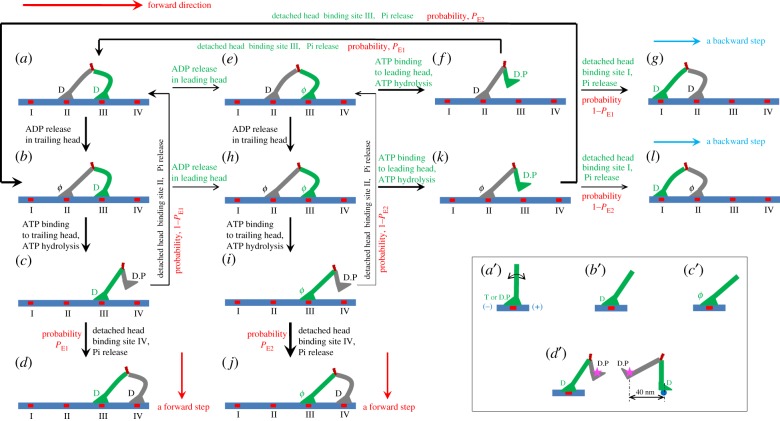
Model of chemomechanical coupling of myosin-V at low ATP. (*a*–*l*) Schematic illustrations of the chemomechanical coupling pathway (see text for detailed description). The thickness of each arrow represents the magnitude of the transition rate or probability under no load. Inside box: (*a′*–*c′*) orientations of the neck domain relative to motor domain bound to actin filament in different nucleotide states; (*d′*) the relative orientation of the two heads in the equilibrium state with one head bound to actin and the other head detached from the actin, with the right panel corresponding to the side view of the left panel. Stars represent the position of the gold particle labelled to the head used in the experiments of Andrecka *et al*. [31].

The model for the chemomechanical coupling of myosin-V was proposed previously [21], which is set up on the basis of the following experimental evidence and arguments. (i) Myosin head in ADP or nucleotide-free (*ϕ*) state has a strong affinity for actin, while in ATP or ADP.Pi state has a weak affinity [22–26]. Moreover, it is argued that after ATP binding there is a very short time period (of the order of microseconds) when the affinity of the ATP-head to the local binding site on actin (denoted by *E*_w1_) is weaker than that to other binding sites (denoted by *E*_w2_) [21]. As explained previously [21,27], the presence of this very short time period is due to the structural change of the actin monomer induced by its strong interaction with myosin head in ADP and *ϕ* states [28–30]. (ii) The orientation of the neck domain of the myosin head relative to its motor domain bound to actin depends on the nucleotide state. In ATP or ADP.Pi state, the neck has random orientations (figure 1*a*′) [32–38]. In ADP state, the neck has a fixed orientation, tilting forward (the plus end of actin) (figure 1*b*′) [25,33,38–42]. In *ϕ* state, the neck tilts forward further (figure 1*c*′) [40–42]. (iii) It is argued that the residue elements connecting the neck and coiled-coil stalk behave elastically, having a small torsional/bendable elastic stiffness, rather than behave completely flexibly, having zero torsional/bendable stiffness. When two heads are not bound to actin or when one head is bound to actin and the other head is detached from actin, the nonzero torsional/bendable elastic stiffness of the residue elements would keep the two heads in a definite relative position and orientation (figure 1*d*′, termed as equilibrium conformation or state), which is in accord with the available experimental and structural data [43–47]. This equilibrium state of the dimer with one head binding to actin filament dictates that the detached head would fluctuate on the left side of the actin-bound head or the actin filament, implying that during processive stepping when one head detaches from the actin filament it always fluctuates temporarily on the left side of the filament, explaining the experimental data of Andrecka *et al*. [31].

The chemomechanical coupling pathway at low ATP is illustrated in figure 1. At low ATP, ADP release and ATP binding are rate-limiting steps of the ATPase activity. Let us begin the chemomechanical coupling cycle with both heads in ADP state binding strongly to actin (figure 1*a*). The rate constant of ADP release from the leading head is much smaller than that from the trailing head (see next section).

First, consider ADP release from the trailing head (figure 1*b*). After ATP binding, the trailing head detaches easily from site II by overcoming the very weak affinity *E*_w1_ and moves to the equilibrium position (figure 1*c*). Then, by overcoming energy (*E*_E_) of retaining the detached head in the equilibrium position and orientation relative to the actin-bound head, the detached head can bind (with probability *P*_E1_) to site IV with affinity *E*_w2_, releasing Pi (figure 1*d*). Alternatively, by overcoming energy *E*_E_ and energy (*E*_B_) of bending the neck of the actin-bound ADP-head, the detached head can rebind (with probability 1 − *P*_E1_) to site II with affinity *E*_w2_, releasing Pi (figure 1*a*) (noting that after ATP binding site II returns elastically to the normally unchanged conformation in a time of the order of microseconds). From figure 1*a*–*d*, a forward step was made. Second, consider in figure 1*a* ADP release from the leading head (figure 1*e*). After ATP binding, the leading head detaches easily from site III by overcoming *E*_w1_ and moves to the equilibrium position (figure 1*f*). Then, by overcoming energy *E*_E_, the detached head can rebind (with probability *P*_E1_) to site III with affinity *E*_w2_, releasing Pi (figure 1*a*). Alternatively, by overcoming energy *E*_E_ and energy *E*_B_, the detached head can bind (with probability 1 − *P*_E1_) to site I with affinity *E*_w2_, releasing Pi (figure 1*g*). From figure 1*a* to 1*g*, a backward step was made.

In figure 1*b*, ADP can also release from the leading head before ATP binding to the trailing head (figure 1*h*). In figure 1*e*, ADP can also release from the trailing head before ATP binding to the leading head (figure 1*h*). From figure 1*h*, after ATP binding to the trailing head, the head detaches easily from site II by overcoming *E*_w1_ and moves to the equilibrium position (figure 1*i*). Then, by overcoming energy *E*_E_, the detached head can bind (with probability *P*_E2_) to site IV with affinity *E*_w2_, releasing Pi (figure 1*j*). Alternatively, by overcoming energy *E*_E_ and energy (*E*_B_*) of bending the neck of the actin-bound *ϕ*-head, the detached head can also rebind (with probability 1 − *P*_E2_) to site II with affinity *E*_w2_, releasing Pi (figure 1*e*). From figure 1*b*–*j*, a forward step was made. From figure 1*h*, after ATP binding to the leading head, the head detaches easily from site III by overcoming *E*_w1_ and moves to the equilibrium position (figure 1*k*). Then, by overcoming energy *E*_E_, the detached head can rebind (with probability *P*_E2_) to site III with affinity *E*_w2_, releasing Pi (figure 1*b*). Alternatively, by overcoming energy *E*_E_ and energy *E*_B_*, the detached head can also bind (with probability 1 − *P*_E2_) to site I with affinity *E*_w2_, releasing Pi (figure 1*l*). From figure 1*e* to 1*l*, a backward step was made.

**Figure 2.**
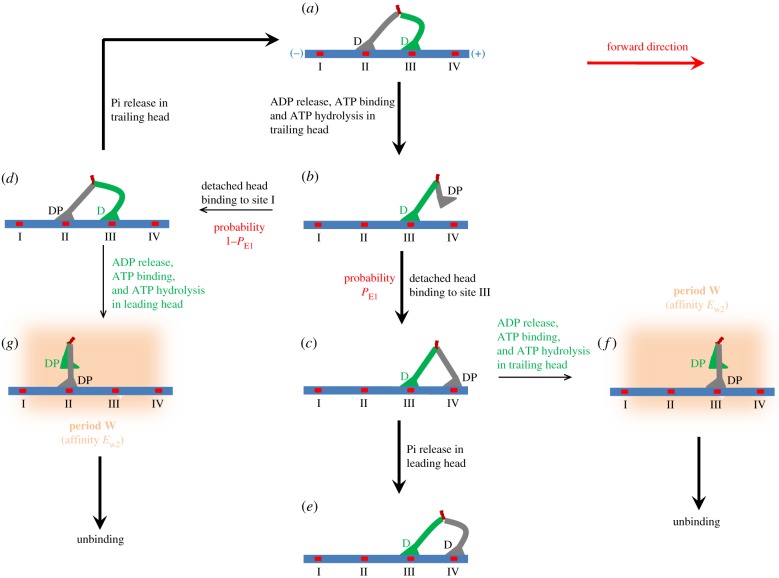
Model of chemomechanical coupling and unbinding of myosin-V at saturating ATP. (*a*–*g*) Schematic illustrations of the occurrence of the weak affinity state with myosin-V binding weakly to actin (see text for detailed descriptions). The thickness of each arrow represents the magnitude of the transition probability under no load.

In figure 1, we only illustrate the chemomechanical coupling of the motor without consideration of the unbinding of the motor from actin. In figure 2, we illustrate the unbinding of the motor during its processive movement. For simplicity, we focus only on saturating ATP in figure 2. Let us still begin the chemomechanical coupling cycle with both ADP-heads binding strongly to actin (figure 2*a*). After ADP release from the trailing head and then ATP binding, the head detaches from site II and moves to the equilibrium position (figure 2*b*). Then, the detached head can either bind (with probability *P*_E1_) to site IV (figure 2*c*) or bind (with probability 1 − *P*_E1_) to site II (figure 2*d*). In figure 2*c*, Pi release in the leading head can take place rapidly before ADP release from the trailing head (figure 2*e*). Occasionally, ADP release in the trailing head can also take place before Pi release from the leading head (figure 2*f*). In figure 2*f*, during the time period (Period W) before Pi release in the actin-bound head takes place, the motor can unbind from actin by overcoming the weak affinity *E*_w2_. In figure 2*d*, Pi release in the trailing head can take place rapidly before ADP release from the leading head (figure 2*a*). Occasionally, ADP release in the leading head can also take place before Pi release from the trailing head (figure 2*g*). In figure 2*g*, during the time period (Period W) before Pi release in the actin-bound head takes place, the motor can unbind from actin by overcoming the weak affinity *E*_w2_. It is mentioned here that in figure 2*a* ADP release and then ATP binding can also take place occasionally in the leading head (not drawn here). If this case occurs, the motor can either make a backward step or make no movement, and occasionally Period W can occur, during which the motor unbinds from actin.

Besides the unbinding during Period W, the motor also has a small probability to unbind during other periods in a chemomechanical coupling cycle (see Results).

### 2.2. Force-independent rate constants of ATPase activity

As done in optical-trapping experiments [5,6,8,9], consider an external force, *F*, acting on the coiled-coil stalk that connects the two necks of the two heads. Here, *F* is defined to be positive, namely, *F* > 0 when it is in the backward direction.

As done in previous work [21], it is proposed that the rate constants of the ATPase activity of the two heads are independent of *F* in the range used in the optical-trapping experiments. It is proposed, however, that the bending of the neck has a large effect on the rate constant of ADP release (the rate-limiting step of the ATPase activity) of the myosin head. In D-D state with both heads in ADP state (e.g. figure 1*a*), the bending of the neck of the leading head induces severe deformations of the head and its nucleotide-binding site. Thus, the rate constant ($k_{D}^{\left(-\right)}$) of ADP release of the leading head is much smaller than that ($k_{D}^{\left(+\right)}$) of the trailing head that has little deformation. In ϕ-D state with the trailing head in *ϕ* state and the leading head in ADP state (e.g. figure 1*b*), the further forward rotation of the neck of the trailing head alleviates the deformation of the leading head relative to that in D-D state. Thus, the rate constant ($k_{D{\;}^{*}}^{\left(-\right)}$) of ADP release of the leading head in ϕ-D state is larger than $k_{D}^{\left(-\right)}$ in D-D state but is smaller than $k_{D}^{\left(+\right)}$. We take $k_{D^{*}}^{\left(-\right)}=C_{D}k_{D}^{\left(-\right)}$, with *C*_D_ > 1. Additionally, both heads have the same rate constant of Pi release, denoted by *k*_P_, and the same second-order rate constant of ATP binding, denoted by *k*_b_. Since after ATP binding the ATP hydrolysis takes place very rapidly, for simplicity, we take the rate constant of ATP hydrolysis to be infinitely large.

## 3. Results and discussion

### 3.1. Velocity, stepping ratio and dwell time with consideration of only ATP-dependent stepping

Since the rate constant of Pi release stimulated by actin is much larger than that of ADP release, for approximation, in this and next sections to study the velocity, stepping ratio and dwell time we take *k*_P_ to be infinitely large.

In our model, probabilities *P*_E1_ and *P*_E2_, as defined in figure 1, are independent of ATP concentration. The expressions for force dependences of *P*_E1_ and *P*_E2_ can be derived as follows. Under the external force *F*, the rate for the detached head to transit from the equilibrium state (e.g. figure 2*b*) to the state binding to the front binding site on actin (e.g. figure 2*c*) can be written as *k*_F_ = *C* exp(− *βFd*^(+)^), where *C* is a constant independent of *F*, *β*^−1^ = *k*_B_*T* is the thermal energy, and *d*^(+)^ is the characteristic distance for the transition. The rate for the detached head to transit from the equilibrium state (e.g. figure 2*b*) to the state binding to the rear binding site on actin (e.g. figure 2*d*) can be written as *k*_R_ = *C* exp(− *βE*_B_)exp(*βFd*^(−)^), where *E*_B_ is the bending energy of the neck of the actin-bound ADP-head when the detached head binds to the rear binding site on actin, as defined above, and *d*^(−)^ is the characteristic distance for the transition. Probability *P*_E1_ can be calculated with *P*_E1_ = *k*_F_/(*k*_F_ + *k*_R_). Substitution of *k*_F_ and *k*_R_ into the above equation yields

3.1 $$P_{E1}=\frac{\exp(\beta E_{B})\exp(-\alpha F)}{\exp(\beta E_{B})\exp(-\alpha F)+1},$$

where *α* = *β*(*d*^(+)^ + *d*^(−)^) is independent of *F*. Similarly, probability *P*_E2_ has the form

3.2 $$P_{E2}=\frac{\exp(\beta E_{B}{\;}^{*})\exp(-\alpha {\;}^{*}F)}{\exp(\beta E_{B}{\;}^{*})\exp(-\alpha {\;}^{*}F)+1},$$

where *E*_B_* is the bending energy of the neck of the actin-bound *ϕ*-head when the detached head binds to the rear binding site on actin, as defined above, and α* = *β*(d*^(+)^ + d*^(−)^) is independent of *F*, with d*^(+)^ and d*^(−)^ being the characteristic distances for the detached head to transit from the equilibrium state (e.g. figure 1*i*) to the states binding to the front and rear binding sites on actin, respectively. Considering that the neck of the actin-bound *ϕ*-head tilts forward more than that of the ADP head, *d*^(+)^ should be larger than d*^(+)^ whereas *d*^(−)^ should be smaller than d*^(−)^. Thus, for approximation, we have $d{*}^{\left(+\right)}+d{*}^{\left(-\right)}\approx d^{\left(+\right)}+d^{\left(-\right)}$ and α* ≈ *α*.

First, we focus on saturating ATP. From figure 2, the stepping ratio of the motor can be calculated by $r=P_{E1}k_{D}^{\left(+\right)}/[(1-P_{E1})k_{D}^{\left(-\right)}]$. Substitution of equation (3.1) into the above equation yields

3.3 $$r=r_{0}^{\left(1-F/F_{S}\right)},$$

where $r_{0}=(k_{D}^{\left(+\right)}/k_{D}^{\left(-\right)})\exp(\beta E_{B})$ is the stepping ratio at *F* = 0 and *F*_S_ = ln(*r*_0_)/*α* is the stall force. With equation (3.3), equation (3.1) can be rewritten as

3.4 $$P_{E1}=\frac{r_{0}^{\left(1-F/F_{S}\right)}}{r_{0}^{\left(1-F/F_{S}\right)}+k_{D}^{\left(+\right)}/k_{D}^{\left(-\right)}}.$$

The velocity of the motor can be calculated by

3.5 $$v=[P_{E1}k_{D}^{\left(+\right)}-(1-P_{E1})k_{D}^{\left(-\right)}]d,$$

where *d* = 36 nm is the step size. The mean dwell time between two mechanical steps can be written as [21]

3.6 $$T_{d}=\frac{1}{k_{D}^{\left(+\right)}P_{E1}+k_{D}^{\left(-\right)}(1-P_{E1})}.$$

Then, we focus on non-saturating ATP. Based on the pathway of figure 1, it is difficult to obtain an exactly analytical solution to the dynamics. Here, we present an approximately analytical solution. For simplicity of treatment, the overall ATPase rate of the trailing head can be approximately written as

3.7 $$k^{\left(+\right)}=\frac{k_{D}^{\left(+\right)}k_{b}[\text{ATP}]}{k_{D}^{\left(+\right)}+k_{b}[\text{ATP}]}.$$

The overall ATPase rate of the leading head can be approximately written as [21]

3.8 $$k^{\left(-\right)}=(1-P_{\phi })\frac{k_{D}^{\left(-\right)}k_{b}[\text{ATP}]}{k_{D}^{\left(-\right)}+k_{b}[\text{ATP}]}+P_{\phi }\frac{k_{D*}^{\left(-\right)}k_{b}[\text{ATP}]}{k_{D*}^{\left(-\right)}+k_{b}[\text{ATP}]},$$

where *P_ϕ_* is the probability of ADP release in the leading head when the trailing head is nucleotide free, which can be calculated by

3.9 $$P_{\phi }=\frac{k_{D*}^{\left(-\right)}}{k_{D*}^{\left(-\right)}+k_{b}[\text{ATP}]}.$$

After ATP binding to the trailing head, the occurrence probability of the equilibrium state with the actin-bound head bound with ADP (e.g. figure 1*c*) is denoted by $P_{D}^{\left(+\right)}$ and the occurrence probability of the equilibrium state with the actin-bound head being nucleotide free (e.g. figure 1*i*) is then $1-P_{D}^{\left(+\right)}$. $P_{D}^{\left(+\right)}$ can be calculated by

3.10 $$P_{D}^{\left(+\right)}=\frac{k_{b}[\text{ATP}]}{k_{D^{*}}^{\left(-\right)}+k_{b}[\text{ATP}]}.$$

After ATP binding to the leading head, the occurrence probability of the equilibrium state with the actin-bound head bound with ADP (e.g. figure 1*f*) is denoted by $P_{D}^{\left(-\right)}$ and the occurrence probability of the equilibrium state with the actin-bound head being nucleotide free (e.g. figure 1*k*) is then $1-P_{D}^{\left(-\right)}$. $P_{D}^{\left(-\right)}$ can be calculated by

3.11 $$P_{D}^{\left(-\right)}=\frac{k_{b}[\text{ATP}]}{k_{D}^{\left(+\right)}+k_{b}[\text{ATP}]}.$$

Thus, after ATP binding to the trailing head, the overall probability of a forward stepping of the motor has the form

3.12 $$P_{\mathrm{EF}}=P_{D}^{\left(+\right)}P_{E1}+(1-P_{D}^{\left(+\right)})P_{E2}.$$

After ATP binding to the leading head, the overall probability of a backward stepping of the motor has the form

3.13 $$P_{\mathrm{EB}}=P_{D}^{\left(-\right)}(1-P_{E1})+(1-P_{D}^{\left(-\right)})(1-P_{E2}).$$

In equations (3.12) and (3.13), *P*_E1_ can be calculated with equation (3.4). With equations (3.1)–(3.4), equation (3.2) for *P*_E2_ can be rewritten as

3.14 $$P_{E2}=\frac{C_{r}r_{0}^{\left(1-F/F_{S}\right)}}{C_{r}r_{0}^{\left(1-F/F_{S}\right)}+k_{D}^{\left(+\right)}/k_{D}^{\left(-\right)}},$$

where $C_{r}=\exp[\beta (E_{B}*-E_{B})]$. Since *E*_B_* > *E*_B_, we have *C*_r_ ≫ 1. As done before [21], we fix *C*_r_ = 100, equivalent to *E*_B_* – *E*_B_ = 4.6 *k*_B_*T*.

The stepping ratio of the motor can be approximately calculated by

3.15 $$r=\frac{P_{\mathrm{EF}}k^{\left(+\right)}}{P_{\mathrm{EB}}k^{\left(-\right)}}.$$

The velocity of the motor can be approximately calculated by

3.16 $$v=(P_{\mathrm{EF}}k^{\left(+\right)}-P_{\mathrm{EB}}k^{\left(-\right)})d.$$

The mean dwell time between two mechanical steps can be approximately calculated by

3.17 $$T_{d}=\frac{1}{P_{\mathrm{EF}}k^{\left(+\right)}+P_{\mathrm{EB}}k^{\left(-\right)}}.$$

From equations (3.7) to (3.17), it is noted that at saturating ATP, equations (3.15), (3.16) and (3.17) reduce to equations (3.3), (3.5) and (3.6), respectively.

**Figure 3.**
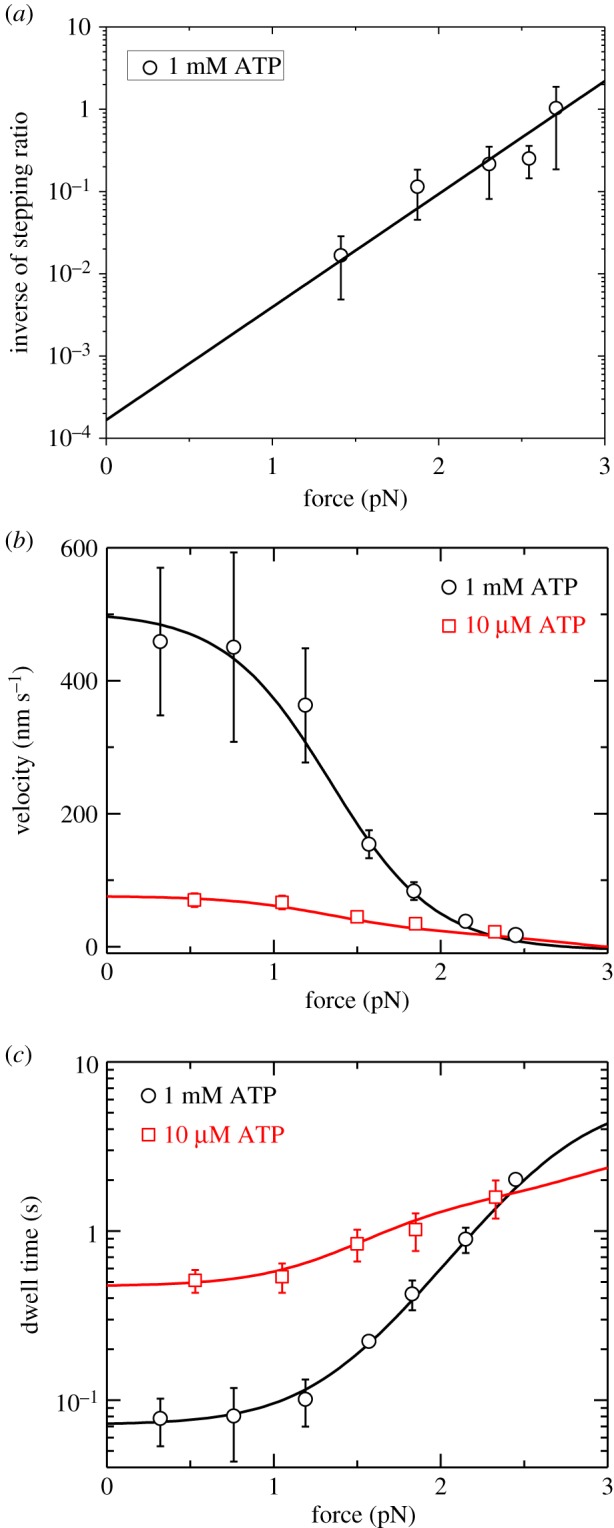
Results for dynamics of chick brain myosin-V under experimental conditions of Uemura *et al*. [6]. Lines are theoretical data with consideration of only ATP-dependent stepping. Symbols are experimental data from Uemura *et al*. [6,49]. (*a*) Force dependence of inverse of stepping ratio at saturating ATP (1 mM). (*b*) Force dependence of velocity at low and saturating ATP concentrations. (*c*) Force dependence of mean dwell time at low and saturating ATP concentrations.

As shown before [21,48], using the above equations the available single-molecule data on force dependences of stepping ratio *r*, velocity *v* and mean dwell time *T*_d_ at different ATP concentrations can be reproduced well with adjustable parameters $k_{D}^{\left(+\right)}$, $k_{D}^{\left(-\right)}$, *r*_0_, *F*_S_, *k*_b_ and *C*_D_. For example, with $k_{D}^{\left(+\right)}=14\;{\text{s}}^{-1}$, $k_{D}^{\left(-\right)}=0.16\;{\text{s}}^{-1}$, *k*_b_ = 0.25 µM^−1^s^−1^, *r*_0_ = 6000, *F*_S_ = 2.75 pN and *C*_D_ = 6 (table 1), the single-molecule data of Uemura *et al*. [6] on force dependences of *r*, *v* and *T*_d_ at both saturating (1 mM) and low (10 µM) ATP concentrations for chick brain myosin-V can be reproduced well (figure 3) (electronic supplementary material).

**Table 1.** Parameter values for different myosin-V (MV) motors under different experimental conditions. Symbol ‘—’ denotes that the value is not required in the calculation.

parameter	chick brain MV under condition of Uemura *et al*. [6]	chick brain MV under condition of Gebhardt *et al*. [9]	murine MV under condition of Zhang *et al*. [7]	chick brain MV under condition of Clemen *et al*. [8]
$k_{D}^{\left(+\right)}$ (s^−1^)	14 ± 1	8 ± 1	13.4 ± 0.8	10.3 ± 0.6
$k_{D}^{\left(-\right)}$ (s^−1^)	0.16 ± 0.02	0.07 ± 0.03	—	0.13 ± 0.04
*k*_b_ (μM^−1^s^−1^)	0.25 ± 0.04	1.8 ± 0.2	0.42 ± 0.05	—
*r*_0_	6000 ± 2000	6000^a^	—	1000 ± 400
*F*_S_ (pN)	2.75 ± 0.06	2.75^a^	—	4 ± 0.5
*C*_D_	6 ± 1.2	6^a^	—	—
$k_{s0}^{\left(-\right)}$ (s^−1^)	—	1.4 ± 0.2	—	0.095 ± 0.016
$k_{s0}^{\left(+\right)}$ (s^−1^)	—	0.13 ± 0.05	—	0^e^
*F*_d_ (pN)	—	4.6 ± 0.3	—	4.6^c^
*k*_P_ (s^−1^)	—	82^b^	280 ± 20	82 ± 8
*ɛ*_s0_ (s^−1^)	—	0.06^b^	0.06 ± 0.008	0.06^d^

^a^Values are taken to be the same as those under condition of Uemura *et al*. [6].

^b^Values are taken to be the same as those under condition of Clemen *et al*. [8].

^c^Value is taken to be the same as that under condition of Gebhardt *et al*. [9].

^d^Value is taken to be the same as that under condition of Zhang *et al*. [7].

^a,b,c,d,e^Values are not adjustable in fitting the experimental data.

### 3.2. Velocity with consideration of both ATP-dependent and ATP-independent steppings

In the above section, we have only considered the ATP-dependent stepping to study the motor dynamics, which is applicable to the case in the range of *F* smaller than the stall force under some experimental conditions, e.g. under the conditions of Uemura *et al*. [6]. In this section, we consider both the ATP-dependent and ATP-independent steppings.

In our model, the ATP-independent stepping arises from the detachment of one head in strong actin-binding state (*ϕ* or ADP state) from actin when the other head is binding fixedly to the actin. Under the external force *F*, the rate of the leading head in strong actin-binding state to detach from actin can be written as

3.18 $$k_{s}^{\left(-\right)}=k_{s0}^{\left(-\right)}\exp\left(\frac{F}{F_{d}^{\left(-\right)}}\right),$$

where $k_{s0}^{\left(-\right)}$ is detaching rate of the leading head under no external force on the motor and $F_{d}^{\left(-\right)}$ is the characteristic detachment force. Similarly, the rate of the trailing head in strong actin-binding state to detach from actin can be written as

3.19 $$k_{s}^{\left(+\right)}=k_{s0}^{\left(+\right)}\exp\left(-\frac{F}{F_{d}^{\left(+\right)}}\right),$$

where $k_{s0}^{\left(+\right)}$ is detaching rate of the trailing head under no external force on the motor and $F_{d}^{\left(+\right)}$ is the characteristic detachment force. For approximation, we take $F_{d}^{\left(+\right)}=F_{d}^{\left(-\right)}=F_{d}$. Since in the state with two heads binding strongly to actin (e.g. figure 2*a*) the internal force arising from the bending of the necks acts on the two heads in different directions, the detaching rates $k_{s0}^{\left(-\right)}$ for the leading head and $k_{s0}^{\left(+\right)}$ for the trailing head would have different values.

As in the case of the head bound weakly to actin, assuming that the neck of the detached head in any nucleotide state also has random orientations, it is noted that by considering both the ATP-dependent and ATP-independent steppings, equation (3.15) for stepping ratio, equation (3.16) for velocity and equation (3.17) for mean dwell time at non-saturating ATP can be replaced by the following equations:

3.20 $$r=\frac{P_{\mathrm{EF}}(k^{\left(+\right)}+k_{s}^{\left(+\right)})}{P_{\mathrm{EB}}(k^{\left(-\right)}+k_{s}^{\left(-\right)})},$$

3.21 $$v=[P_{\mathrm{EF}}(k^{\left(+\right)}+k_{s}^{\left(+\right)})-P_{\mathrm{EB}}(k^{\left(-\right)}+k_{s}^{\left(-\right)})]d$$

3.22 $$\text{and}\;T_{d}=\frac{1}{P_{\mathrm{EF}}(k^{\left(+\right)}+k_{s}^{\left(+\right)})+P_{\mathrm{EB}}(k^{\left(-\right)}+k_{s}^{\left(-\right)})}.\;$$

**Figure 4.**
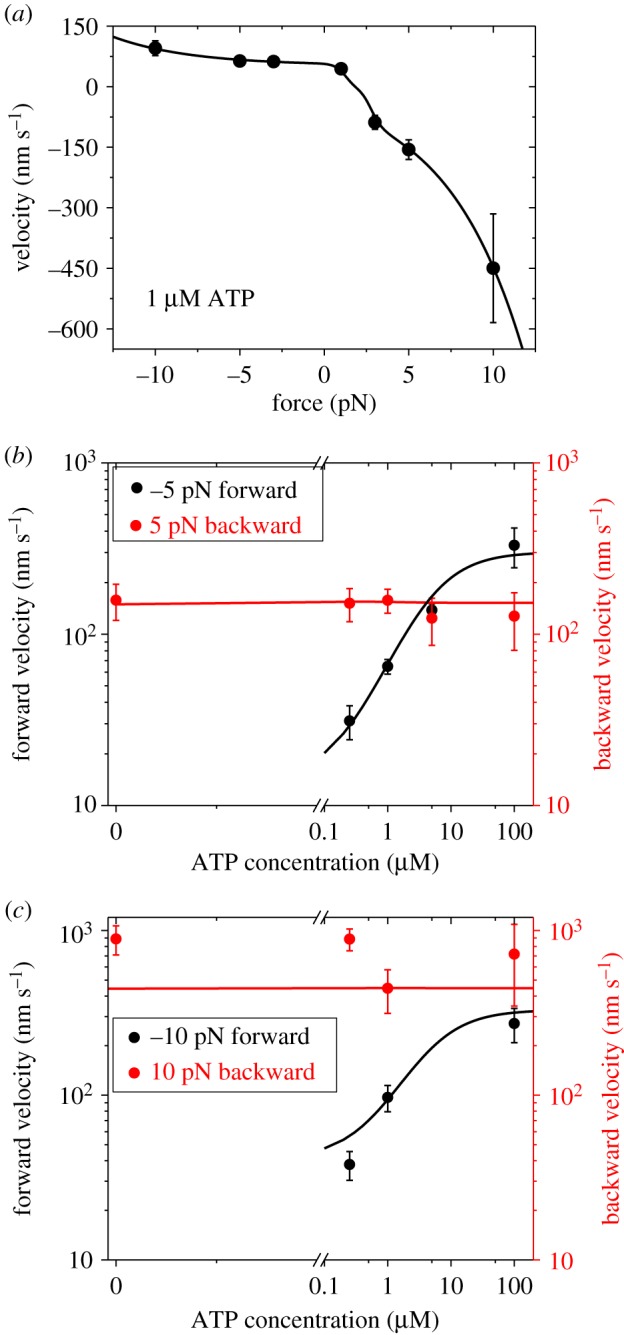
Results for dynamics of chick brain myosin-V under experimental conditions of Gebhardt *et al*. [9]. Lines are theoretical data with consideration of both ATP-dependent and ATP-independent steppings. Symbols are experimental data from Gebhardt *et al*. [9]. (*a*) Velocity versus force at 1 µM ATP. (*b*) Velocity versus ATP concentration at –5 pN (forward) and 5 pN (backward) forces. (*c*) Velocity versus ATP concentration at –10 pN (forward) and 10 pN (backward) forces.

Now, we use the above equations to fit the single-molecule data of Gebhardt *et al*. [9] on the dependence of velocity upon force and ATP concentration for chick brain myosin-V. We use the same values of parameters *r*_0_, *F*_S_ and *C*_D_ as those used in figure 3 (table 1). With adjustable parameters $k_{D}^{\left(+\right)}=9\;{\text{s}}^{-1}$, $k_{D}^{\left(-\right)}=0.08\;{\text{s}}^{-1}$, *k*_b_ = 1.8 µM^−1^ s^−1^, $k_{s0}^{\left(-\right)}=1.4\;{\text{s}}^{-1}$ and *F*_d_ = 4.6 pN (table 1), the single-molecule data [9] can be reproduced well (figure 4) (electronic supplementary material).

Intriguingly, the single-molecule data of Gebhardt *et al*. [9] showed that the distribution of dwell time for backward steps under superstall force (*F* > 3 pN) has a biphasic character, namely, the distribution can be fitted well by the sum of two single exponentials with two positive amplitudes. Based on our model, we provide a quantitative explanation of this biphasic character (see appendix A).

### 3.3. Unbinding rate and run length

In our model, during the processive stepping the unbinding of the motor from actin occurs mainly during Period W (figure 2), especially at high ATP concentrations. The expression for the unbinding rate by considering that the unbinding can occur only during Period W can be derived as follows.

Period W comes from the state of the motor with one head in ADP or *ϕ* state binding strongly to actin and the other head in ADP.Pi state binding weakly to actin with affinity *E*_w2_ (e.g. figure 2*c*,*d*). (i) In the state with the trailing head in ADP or *ϕ* state and the leading head in ADP.Pi state (e.g. figure 2*c*), if ADP release and ATP binding in the trailing head take place before Pi release in the leading head, Period W occurs. (ii) In the state with the trailing head in ADP.Pi state and the leading head in ADP or *ϕ* state (e.g. figure 2*d*), if ADP release and ATP binding in the leading head take place before Pi release in the trailing head, Period W also occurs. Since the occurrence of Period W is determined by the rate constant of Pi release relative to that of ADP release, the large rate constant of Pi release, *k*_P_, must be taken into account to study the unbinding rate.

First, we focus on saturating ATP. In one ATPase cycle, the occurrence probability of case (i) can be calculated by $P_{E1}k_{D}^{\left(+\right)}/(k_{D}^{\left(+\right)}+k_{P})$ and the occurrence probability of case (ii) can be calculated by $\left(1-P_{E1})k_{D}^{\left(-\right)}/(k_{D}^{\left(-\right)}+k_{P}\right)$. Thus, the occurrence probability of Period W in one ATPase cycle can be calculated by

3.23 $$P_{w}=P_{E1}\frac{k_{D}^{\left(+\right)}}{k_{D}^{\left(+\right)}+k_{P}}+(1-P_{E1})\frac{k_{D}^{\left(-\right)}}{k_{D}^{\left(-\right)}+k_{P}}.$$

The total ATPase rate can be calculated by

3.24 $$k=k_{D}^{\left(+\right)}+k_{D}^{\left(-\right)}.$$

Supposing that the motor unbinds from actin when Period W occurs, the unbinding rate by considering that the unbinding can occur only during Period W can be calculated by

3.25 $$\varepsilon_{w}=kP_{w}.$$

Then, we focus on non-saturating ATP. In one ATPase cycle, the occurrence probability of case (i) can be approximately calculated by *P*_EF_*k*^(+)^/(*k*^(+)^ + *k*_P_) and the occurrence probability of case (ii) can be approximately calculated by *P*_EB_*k*^(−)^/(*k*^(−)^ + *k*_P_). Thus, the occurrence probability of Period W in one ATPase cycle can be approximately calculated by

3.26 $$P_{w}=P_{\mathrm{EF}}\frac{k^{\left(+\right)}}{k^{\left(+\right)}+k_{P}}+P_{\mathrm{EB}}\frac{k^{\left(-\right)}}{k^{\left(-\right)}+k_{P}}.$$

The total ATPase rate can be approximately calculated by

3.27 $$k=k^{\left(+\right)}+k^{\left(-\right)}.$$

The unbinding rate by considering that the unbinding can occur only during Period W can still be calculated by equation (3.25), but with *k* being calculated by equation (3.27) and *P*_w_ being calculated by equation (3.26).

Besides unbinding during Period W, the motor can also unbind with a small probability during other periods when the motor binds strongly to actin in a chemomechanical coupling cycle. Since in a chemomechanical coupling cycle the motor is almost always in the state with both heads binding strongly to actin, the unbinding during other periods except Period *W* should occur mainly in the period with both heads binding strongly to actin. Thus, the unbinding rate during other periods except Period *W*, which is denoted by *ɛ*_s_, should be approximately a constant value independent of ATP concentration. According to Kramers, theory, the force dependence of *ɛ*_s_ can be written as

3.28 $$\varepsilon_{s}=\varepsilon_{s0}\exp\left(\frac{\left|F\right|}{F_{d}}\right),$$

where *ε*_s0_ is the unbinding rate at *F* = 0 and *F*_d_ is the characteristic unbinding force, as defined in the above section.

The total unbinding rate can be written as

3.29 $$\varepsilon =\varepsilon_{w}+\varepsilon_{s}.$$

The run length can be calculated by

3.30 $$L=\frac{v}{\varepsilon }.$$

**Figure 5.**
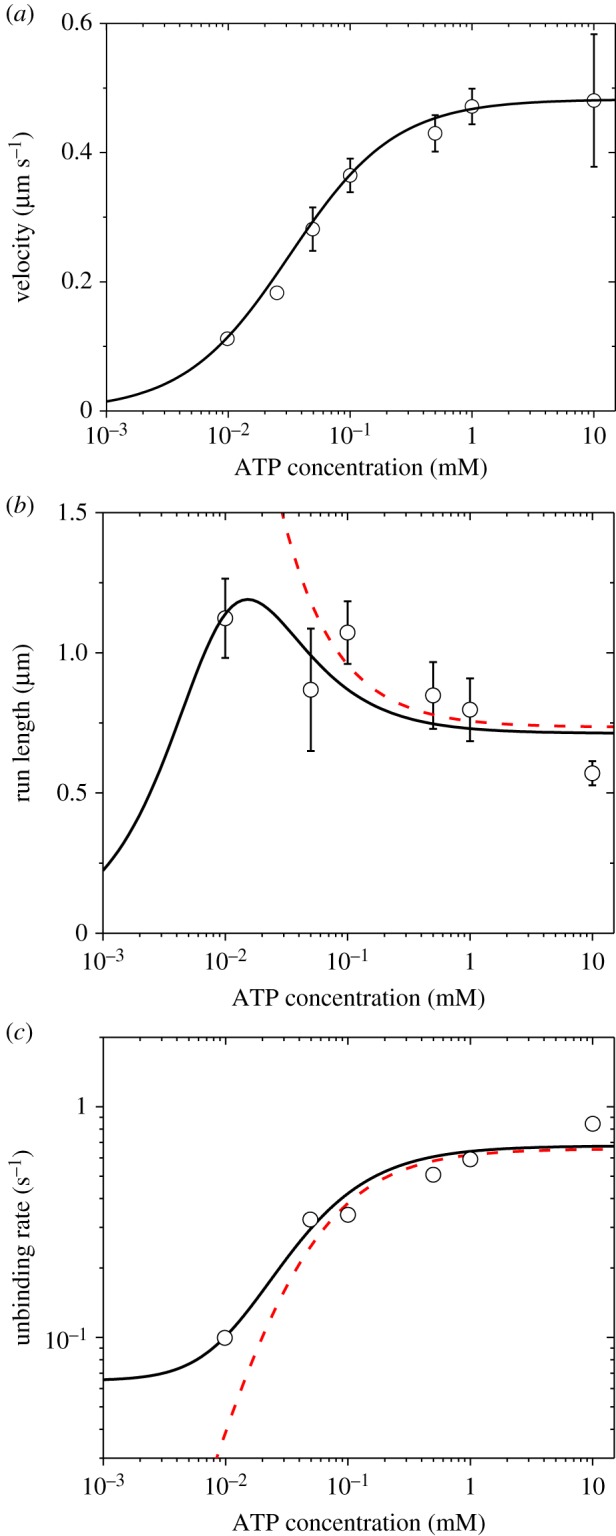
Results for dynamics of murine myosin-V at no load under experimental conditions of Zhang *et al*. [7]. Lines are theoretical data with consideration of only ATP-dependent stepping, with red dashed lines corresponding to the case that the unbinding of the motor can occur only during Period W and black solid lines corresponding to the case that the unbinding can occur during both Period W and other periods. Symbols are experimental data from Zhang *et al*. [7], with the experimental data in (*c*) being calculated from those in (*a*) and (*b*). (*a*) Velocity versus ATP concentration. (*b*) Run length versus ATP concentration. (*c*) Unbinding rate versus ATP concentration.

First, we use the above equations to fit the single-molecule data of Zhang *et al*. [7] on dependences of velocity *v*, run length *L* and unbinding rate *ɛ* upon ATP concentration under no external force for murine myosin-V. With above-fitted values for chick brain myosin-V under experimental conditions of Uemura *et al*. [6] and Gebhardt *et al*. [9] (table 1), we obtain *P*_E1_ ≈ 1 and *P*_E2_ ≈ 1 under *F* = 0. Thus, to fit the experimental data of Zhang *et al*. [7] on dependence of *v* upon ATP concentration under *F* = 0, for approximation, only parameters $k_{D}^{\left(+\right)}$ and *k*_b_ are required. Moreover, to fit the experimental data on dependence of *L* and *ɛ* upon ATP concentration under *F* = 0, two additional parameters *k*_P_ and *ɛ*_s0_ are required. With adjustable parameters $k_{D}^{\left(+\right)}=13.4\;{\text{s}}^{-1}$, *k*_b_ = 0.42 µM^−1^s^−1^, *k*_P_ = 280 s^−1^ and *ɛ*_s0_ = 0.06 s^−1^ (table 1), the single-molecule data [7] can be reproduced well (figure 5) (electronic supplementary material). For comparison, in figure 5*b*,*c* (dashed lines), we also show the theoretical results calculated by considering that the unbinding can occur only during Period W. Interestingly, from figure 5*c*, it is seen that the unbinding rate decreases with the decrease of ATP concentration, resulting in the run length increasing with the decrease of ATP concentration in the range of [ATP] > 12 µM (Figure 5*b*). However, with the further decrease of ATP concentration in the range of [ATP] < 12 µM, the run length decreases when the total unbinding is considered (solid line in Figure 5*b*). This is because in the range of [ATP] < 12 µM, as [ATP] decreases the total unbinding rate becomes nearly levelled off (solid line in figure 5*c*) and the sensitive decrease of *v* with the decrease of [ATP] (figure 5*a*) results in the decrease of the run length.

**Figure 6.**
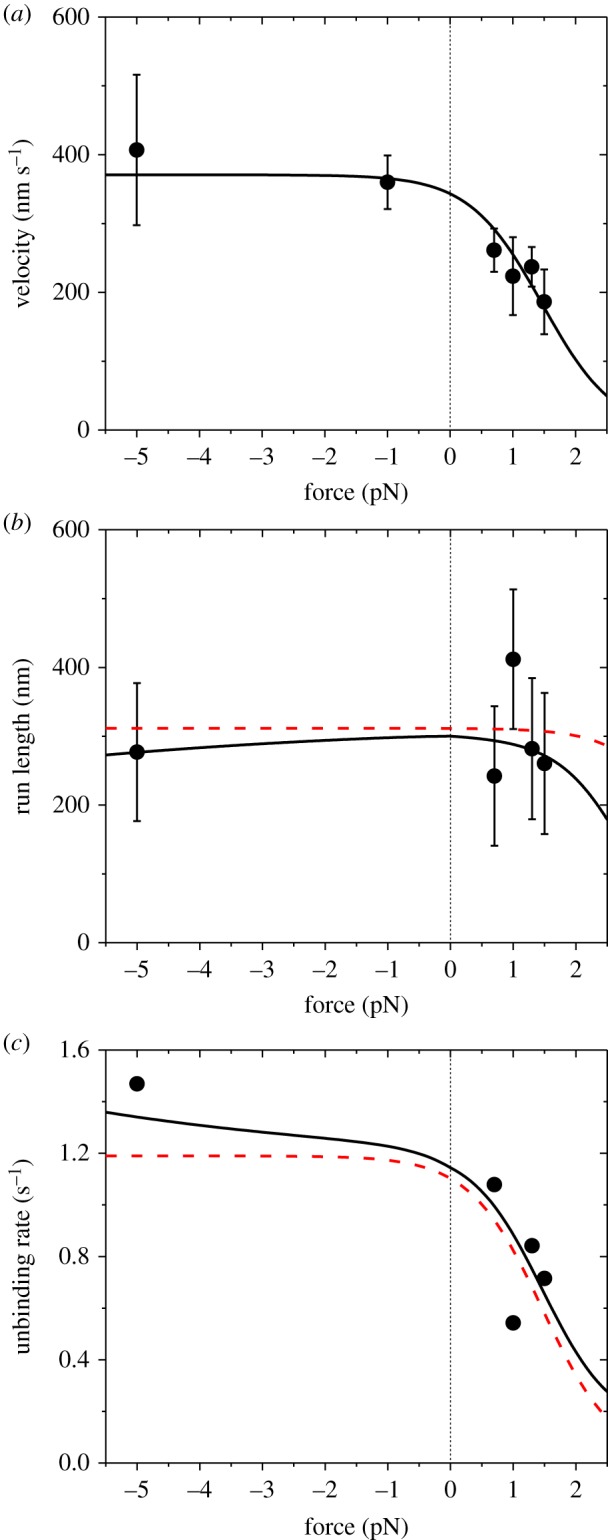
Results for dynamics of chick brain myosin-V at saturating ATP under experimental conditions of Clemen *et al*. [8]. Lines are theoretical data with consideration of only ATP-dependent stepping, with red dashed lines corresponding to the case that the unbinding of the motor can occur only during Period W and black solid lines corresponding to the case that the unbinding can occur during both Period W and other periods. Symbols are experimental data from Clemen *et al*. [8], with the experimental data in (*c*) being calculated from those in (*a*) and (*b*). (*a*) Force dependence of velocity. (*b*) Force dependence of run length. (*c*) Force dependence of unbinding rate.

Then, we fit the single-molecule data of Clemen *et al*. [8] on dependences of velocity *v*, run length *L* and unbinding rate *ɛ* upon external force *F* in the range of *F* < 2.5 pN at saturating ATP for chick brain myosin-V. As noted from figure 4, in the range of *F* < 2.5 pN the ATP-independent stepping makes a much smaller contribution to the movement than ATP-dependent stepping. Thus, for approximation, we neglect the ATP-independent stepping here. As in figure 4, we take *F*_d_ = 4.6 pN, and as in figure 5, we take *ɛ*_s0_ = 0.06 s^−1^. With adjustable parameters $k_{D}^{\left(+\right)}=10.3\;{\text{s}}^{-1}$, $k_{D}^{\left(-\right)}=0.13\;{\text{s}}^{-1}$, *r*_0_ = 1000, *F*_S_ = 4 pN and *k*_P_ = 82 s^−1^ (table 1), the single-molecule data [8] can be reproduced well (figure 6) (electronic supplementary material). For comparison, in figure 6*b*,c (dashed lines), we also show the theoretical results calculated by considering that the unbinding can occur only during Period W. From figure 6*a*,*b*, it is seen that both the theoretical and experimental data showed that in the range of *F* = −5 to 1.5 pN the run length is almost independent of *F*, although the velocity decreases evidently with the increase of *F* for *F* > 0. More interestingly, from figure 6*c*, it is seen that under the backward force in the range of *F* < 2.5 pN, the unbinding rate has the characteristic of a catch bond, with the unbinding rate decreasing with the increase of the backward force.

**Figure 7.**
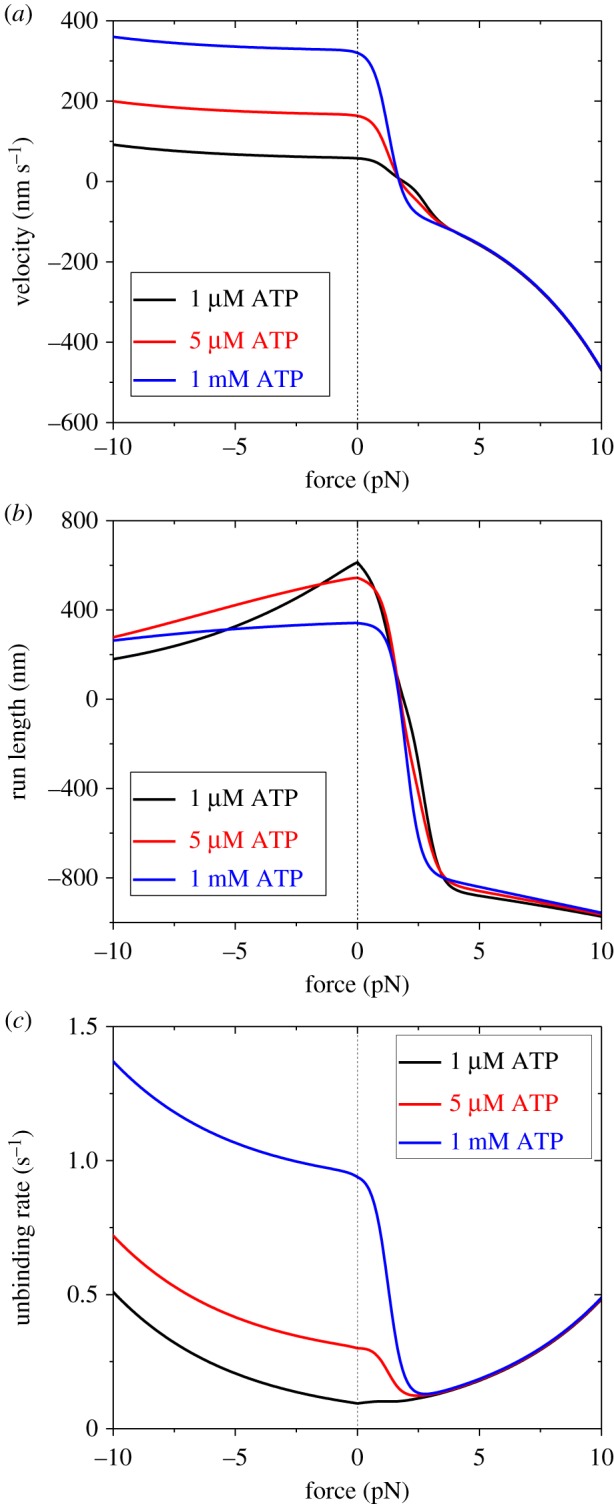
Results for dynamics of chick brain myosin-V under experimental conditions of Gebhardt *et al*. [9]. Lines are predicted theoretical data at different ATP concentrations with consideration of both ATP-dependent and ATP-independent stepping. (*a*) Force dependence of velocity. (*b*) Force dependence of run length. (*c*) Force dependence of unbinding rate.

Up to now, we have fitted the available experimental data on the dynamics of myosin-V. Then, we provide some predicted results, which can be tested easily using single-molecule optical trappings. With parameter values for chick brain myosin-V under experimental conditions of Gebhardt *et al*. [9] (table 1) and by additionally taking *k*_P_ = 82 s^−1^ and *ɛ*_s0_ = 0.06 s^−1^, as given in figure 6, we show the predicted results on force dependences of velocity *v*, run length *L* and unbinding rate *ɛ* at different ATP concentrations (figure 7). Interestingly, from figure 7*c*, it is seen that at very low ATP (1 µM) the unbinding rate has the characteristic of a slip bond for both the forward and backward loads. By contrast, at an ATP concentration that is not very low (e.g. ≥ 5 µM), the unbinding rate has the characteristic of a catch-slip bond for the backward load and the characteristic of a slip bond for the forward load.

**Figure 8.**
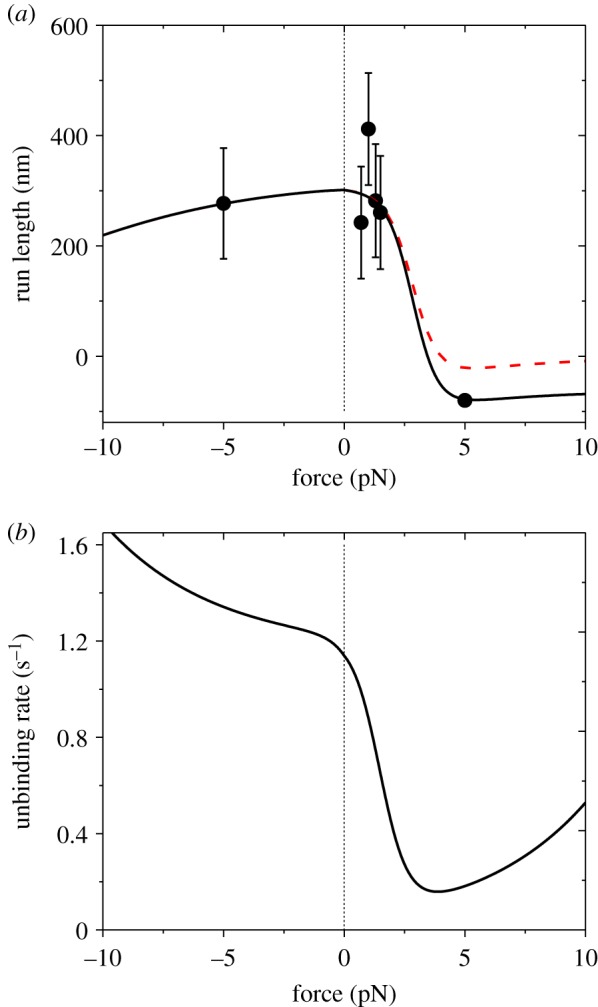
Results for dynamics of chick brain myosin-V at saturating ATP under experimental conditions of Clemen *et al*. [8]. Line are theoretical data, with black solid lines corresponding to the case with consideration of both ATP-dependent and ATP-independent steppings, while the red dashed line corresponding to the case with consideration of only ATP-dependent stepping. Symbols are experimental data from Clemen *et al*. [8]. (*a*) Force dependence of run length. (*c*) Force dependence of unbinding rate.

In figure 6, we show the results in the range of *F* < 2.5 pN at saturating ATP for chick brain myosin-V under experimental conditions of Clemen *et al*. [8]. Now, we give results in the large range of *F*. With consideration of only ATP-dependent stepping, as done in figure 6, the force dependences of run length *L* and unbinding rate *ɛ* are shown in figure 8*a* (dashed line) and figure 8*b*, respectively. With consideration of both ATP-dependent and ATP-independent steppings, we still take *F*_d_ = 4.6 pN as in figure 4. With adjustable parameter $k_{s0}^{\left(-\right)}=0.095\;{\text{s}}^{-1}$, the theoretical data (solid line in figure 8*a*) reproduce the experimental data of Clemen *et al*. [8]. Here, for approximation, we take $k_{s0}^{\left(+\right)}=0$ because $k_{s0}^{\left(+\right)}\ll k_{s0}^{\left(-\right)}$. The predicted results of the unbinding rate versus *F* are shown in figure 8*b*. Note that whether for the case with inclusion of the ATP-independent backward stepping or not, we have the same unbinding rate. From figure 8*b*, we see that at saturating ATP, the unbinding rate has the catch-slip-bond characteristic for the backward load and the slip-bond characteristic for the forward load, as indicated in figure 7*c*.

## 4. Concluding remarks

The dynamics of myosin-V is studied theoretically based on our proposed model. In the studies, both ATP-dependent and ATP-independent steppings are considered. Specifically, the dependences of velocity, run length and unbinding rate upon external force and ATP concentration are studied, giving quantitative explanations of the available single-molecule data and providing predicted results. Interestingly, the results show that the unbinding rate increases with the increase of ATP concentration and levels off at both low and high ATP concentrations. More interestingly, at an ATP concentration that is not very low, the unbinding rate exhibits the catch-slip-bond characteristic under the backward load, with the unbinding rate decreasing rapidly with the increase of the backward load in the range smaller than about 2.5 pN and then increasing slowly with the increase of the backward load. By contrast, under the forward load, the unbinding rate exhibits the slip-bond characteristic. In the future, we should rely on these force and ATP-concentration dependences of velocity and unbinding rate to study theoretically and/or computationally the collective transport by multiple myosin-V motors.

## Supplementary Material

Fitting methods

## Figures and Tables

**Figure 9.**
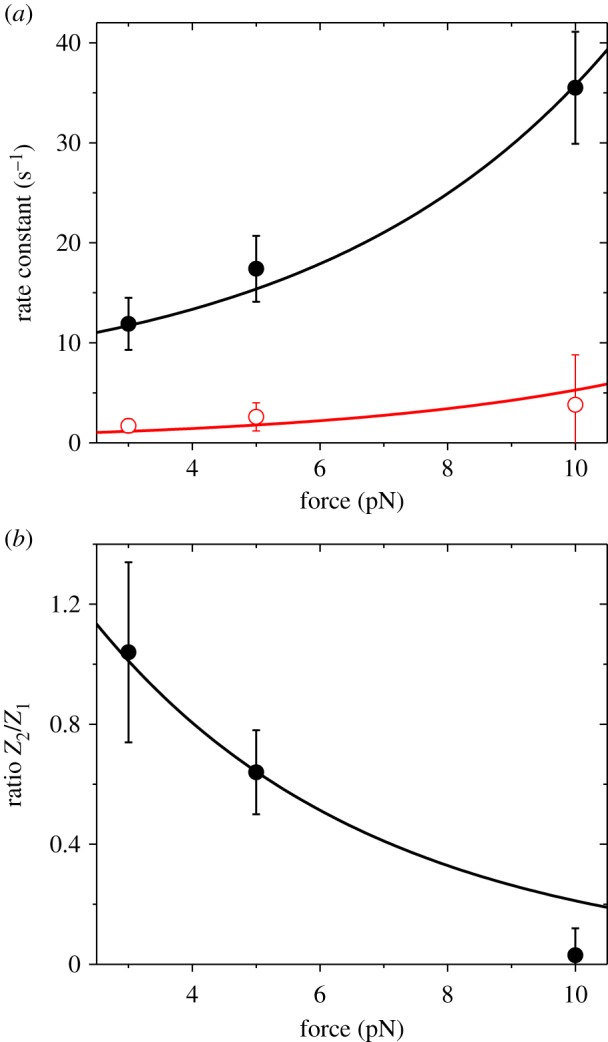
Results for biphasic character of dwell-time distribution for backward steps under superstall force (*F* > 3 pN) under experimental conditions of Gebhardt *et al*. [9]. Lines are theoretical data. Symbols are experimental data from Gebhardt *et al*. [9]. (*a*) Force dependences of rate constants *λ*_1_ and *λ*_2_. (*b*) Force dependence of amplitude ratio *Z*_2_/*Z*_1_.

## Data accessibility

This article does not contain any additional data.

## Appendix A. Biphasic character of dwell-time distribution for backward steps under superstall force

We suppose that there are two strong actin-binding states of a myosin head. One is state S1 and the other is state S2, with state S2 having a slightly larger affinity to actin than state S1. Upon the detached ADP-head or *ϕ*-head binding strongly to actin, the head is initially in state S1. Then, state S1 transits to state S2, with the rate constant of the transition being denoted by $k_{\mathrm{tr}}^{\left(-\right)}$ for the leading head and by $k_{\mathrm{tr}}^{\left(+\right)}$ for the trailing head. Both rate constants $k_{\mathrm{tr}}^{\left(-\right)}$ and $k_{\mathrm{tr}}^{\left(+\right)}$ are independent of *F* in the range used in the optical-trapping experiments. Under the backward force *F*, the rate of the leading head in state S1 to detach from actin is denoted by $k_{s1}^{\left(-\right)}$ and in state S2 is denoted by $k_{s2}^{\left(-\right)}$. Evidently, $k_{s1}^{\left(-\right)}>k_{s2}^{\left(-\right)}$.

Here, we focus on superstall force (*F* > 3 pN). As shown in the main text, under *F* > 3 pN, the average rate of the leading head in strong actin-binding state to detach from actin, $k_{s}^{\left(-\right)}$, is much larger than the rate of the leading head to detach from actin arising from ATP binding, *k*^(−)^. In addition, *P*_E1_ ≈ 0 and *P*_E2_ ≈ 0 under *F* > 3 pN. Thus, the backward stepping rate is approximately equal to $k_{s1}^{\left(-\right)}$ in state S1 and $k_{s2}^{\left(-\right)}$ in state S2. Consequently, the pathway of a backward step under *F* > 3 pN can be described by the following scheme:

In the scheme, S_1_ and S_2_ represent state S1 and state S2 of the leading head, respectively, and B represents the state when the leading head becomes the trailing one, i.e. the transition to B represents that a backward step occurs. Denoting by *P*_S1_, *P*_S2_ and *P*_B_ the probabilities of states S1, S2 and B, respectively, the probability density for the dwell time, *f*(*t*), can be calculated with following differential equations:

A 1 $$\frac{\text{d}P_{S1}}{\text{d}t}=-(k_{\mathrm{tr}}^{\left(-\right)}+k_{s1}^{\left(-\right)})P_{S1},$$

A 2 $$\frac{\text{d}P_{S2}}{\text{d}t}=k_{\mathrm{tr}}^{\left(-\right)}P_{S1}-k_{s2}^{\left(-\right)}P_{S2}$$

A 3 $$\mathrm{and}\;\frac{\text{d}P_{B}}{\text{d}t}=k_{s1}^{\left(-\right)}P_{S1}+k_{s2}^{\left(-\right)}P_{S2},$$

with the initial condition *P*_S1_(*t* = 0) = *a*, *P*_S2_(*t* = 0) = 1 − *a* and *P*_B_(*t* = 0) = 0, where *a* is the probability of the leading head in state S1 at *t* = 0, which can be calculated with

A 4 $$a=\frac{k_{s}^{\left(-\right)}}{k_{\mathrm{tr}}^{\left(+\right)}+k_{s}^{\left(-\right)}}.$$

The force dependences of $k_{s1}^{\left(-\right)}$ and $k_{s2}^{\left(-\right)}$ can be written as

A 5 $$k_{s1}^{\left(-\right)}=k_{s01}^{\left(-\right)}\exp\left(\frac{F}{F_{d}}\right)$$

and

A 6 $$k_{s2}^{\left(-\right)}=k_{s02}^{\left(-\right)}\exp\left(\frac{F}{F_{d}}\right),$$

where *F*_d_ is the characteristic unbinding force, as defined in the main text, and $k_{s01}^{\left(-\right)}>k_{s0}^{\left(-\right)}$ and $k_{s02}^{\left(-\right)}<k_{s0}^{\left(-\right)}$, with $k_{s0}^{\left(-\right)}$ being the mean detaching rate of the leading head in strong actin-binding state at *F* = 0, as defined in the main text.

The probability density for the dwell time can be calculated by *f*(*t*) = d*P*_B_/d*t*. Solving equations (A 1)–(A 3), we finally obtain

A 7 $$f(t)=Z_{1}\lambda_{1}\exp(-\lambda_{1}t)+Z_{2}\lambda_{2}\exp(-\lambda_{2}t),$$

A 8 $$\lambda_{1}=k_{\mathrm{tr}}^{\left(-\right)}+k_{s1}^{\left(-\right)},$$

A 9 $$\lambda_{2}=k_{s2}^{\left(-\right)},$$

A 10 $$Z_{1}=\frac{a(k_{s1}^{\left(-\right)}-k_{s2}^{\left(-\right)})}{k_{\mathrm{tr}}^{\left(-\right)}+k_{s1}^{\left(-\right)}-k_{s2}^{\left(-\right)}}$$

A 11 $$\mathrm{and}\;Z_{2}=\frac{k_{\mathrm{tr}}^{\left(-\right)}+(1-a)(k_{s1}^{\left(-\right)}-k_{s2}^{\left(-\right)})}{k_{\mathrm{tr}}^{\left(-\right)}+k_{s1}^{\left(-\right)}-k_{s2}^{\left(-\right)}}.$$

With equations (A 10) and (A 11), the amplitude ratio has the form

A 12 $$\frac{Z_{2}}{Z_{1}}=\frac{k_{\mathrm{tr}}^{\left(-\right)}+(1-a)(k_{s1}^{\left(-\right)}-k_{s2}^{\left(-\right)})}{a(k_{s1}^{\left(-\right)}-k_{s2}^{\left(-\right)})}.$$

As expected, from equations (A 10) and (A 11) it is seen that *Z*_1_ + *Z*_2_ = 1. Since 0 < *a* ≤ 1 and $k_{s1}^{\left(-\right)}>k_{s2}^{\left(-\right)}$, from equations (A 10) and (A 11) it is noted that *Z*_1_ > 0 and *Z*_2_ > 0. Thus, from equation (A 7) we see that the dwell-time distribution for the backward steps under superstall force (*F* > 3 pN) has the biphasic character, with the sum of two single exponentials, one having a rate constant *λ*_1_ and amplitude *Z*_1_ and the other having a rate constant *λ*_2_ and amplitude *Z*_2_. This biphasic character is consistent with the experimental data of Gebhardt *et al*. [9]. Specifically, using equations (A 5), (A 6), (A 8) and (A 9), with *F*_d_ = 4.6 pN given in table 1 and by adjusting $k_{s01}^{\left(-\right)}=3.5\;{\text{s}}^{-1}$, $k_{s02}^{\left(-\right)}=0.6\;{\text{s}}^{-1}$ and $k_{\mathrm{tr}}^{\left(-\right)}=5\;{\text{s}}^{-1}$, the theoretical data of rate constants *λ*_1_ and *λ*_2_ versus *F* are in quantitative agreement with the experimental data of Gebhardt *et al*. [9] (figure 9*a*). Moreover, using equations (A 4)–(A 6) and (A 12), with above parameter values for $k_{s01}^{\left(-\right)}$, $k_{s02}^{\left(-\right)}$ and $k_{\mathrm{tr}}^{\left(-\right)}$, with *F*_d_ = 4.6 pN and $k_{s0}^{\left(-\right)}=1.4\;{\text{s}}^{-1}$ given in table 1 and by adjusting only $k_{\mathrm{tr}}^{\left(+\right)}=0.16\;{\text{s}}^{-1}$, the theoretical data of amplitude ratio *Z*_2_/ *Z*_1_ are also close to the experimental data [9] (figure 9*b*).
