# Supplementary material for: Dynamics of ATP-dependent and ATP-independent steppings of myosin-V on actin: catch-bond characteristics
Source: J R Soc Interface. 2020 Apr 8;17(165):20200029. doi: 10.1098/rsif.2020.0029 (PMC7211485; doi:10.1098/rsif.2020.0029)
Supplement: Fitting methods [file rsif20200029supp1.pdf]

Supplementary materials

Dynamics of ATP-dependent and ATP-independent steppings of myosin-V on actin: catch-bond characteristics

Ping Xie

Institute of Physics, Chinese Academy of Sciences, Beijing 100190, China

Fitting methods

The methods to fit the experimental data in different figures (Figs. 3 – 6) can be described as follows. In each figure, the values of adjustable parameters with standard errors were determined by least-squares fittings to the experimental data.

Figure 3. Using Eq. (3) and with two adjustable parameters r_0 and F_S, the experimental data of Uemura et al. [6] on force dependence of stepping ratio at saturating ATP can be fitted (Fig. 3a). Using Eq. (5), with fixed parameters r_0 and F_S determined above and with two adjustable parameters $k_D^{(+)}$ and $k_D^{(-)}$, the experimental data of Uemura et al. [6] on force dependence of velocity at saturating ATP (1 mM) can be fitted (Fig. 3b, black line). Using Eq. (16), with fixed parameters r_0, F_S, $k_D^{(+)}$ and $k_D^{(-)}$ determined above and with two adjustable parameters k_b and C_D, the experimental data of Uemura et al. [6] on force dependence of velocity at non-saturating ATP (1 μM) can be fitted (Fig. 3b, red line). Using Eq. (6) and with parameters r_0, F_S, $k_D^{(+)}$ and $k_D^{(-)}$ determined above, the theoretical results on force dependence of dwell time at saturating ATP can be calculated (Fig. 3c, black line). Using Eq. (17) and with parameters r_0, F_S, $k_D^{(+)}$, $k_D^{(-)}$, k_b and C_D determined above, the theoretical results on force dependence of dwell time at 1 μM ATP can be calculated (Fig. 3c, red line).

Figure 4. Using Eq. (21), with the same values of parameters r_0, F_S and C_D as those given in Fig. 3 and with adjustable parameters $k_D^{(+)}$, $k_D^{(-)}$, k_b, $k_{s0}^{(-)}$, $k_{s0}^{(+)}$ and F_d, the experimental data of Gebhardt et al. [9] on force dependence of velocity at 1

μM ATP can be fitted (Fig. 4a). Using Eq. (21) and with the parameters determined above, the theoretical results on dependence of velocity upon ATP concentration under different forces can be calculated (Fig. 4b and c).

Figure 5. Using Eqs. (7) and (16) for $P_{E1} = 1$ and $P_{E2} = 1$ (i.e., $P_{EF} = 1$ and $P_{EB} = 0$) under $F = 0$ and with two adjustable parameter $k_D^{(+)}$ and k_b, the experimental data of Zhang et al. [7] on dependence of velocity upon ATP concentration under $F = 0$ can be fitted (Fig. 5a). Using Eqs. (7), (16) and (25) – (30), with fixed parameters $k_D^{(+)}$ and k_b determined above and with two adjustable parameters k_P and ε_{s0}, the experimental data of Zhang et al. [7] on dependence of run length upon ATP concentration under $F = 0$ can be fitted (Fig. 5b, solid black line). The dashed red line in Fig. 5b was calculated by taking $\varepsilon_{s0} = 0$. Using Eqs. (25) – (29) and with the parameters determined above, the theoretical results on dependence of unbinding rate upon ATP concentration under $F = 0$ can be calculated (Fig. 5c, solid black line). The dashed red line in Fig. 5c was calculated by taking $\varepsilon_{s0} = 0$.

Figure 6. Using Eq. (5) and with adjustable parameters r_0, F_S, $k_D^{(+)}$ and $k_D^{(-)}$, the experimental data of Clemen et al. [8] on force dependence of velocity at saturating ATP can be fitted (Fig. 6a). Using Eq. (5), (23) – (25) and (28) – (30), with fixed parameters r_0, F_S, $k_D^{(+)}$ and $k_D^{(-)}$ determined above, with fixed parameters F_d and ε_{s0} as given in Figs. 4 and 5, respectively, and with one adjustable parameter k_P, the experimental data of Clemen et al. [8] on force dependence of run length at saturating ATP can be fitted (Fig. 6b, solid black line). The dashed red line in Fig. 6b was calculated by taking $\varepsilon_{s0} = 0$. Using Eq. (5) and (25) – (29) and with the parameters determined above, the theoretical results on force dependence of unbinding rate can be calculated (Fig. 6c, solid black line). The dashed red line in Fig. 6c was calculated by taking $\varepsilon_{s0} = 0$.
